# Polymorphisms in *ERCC1* and *XPF* Genes and Risk of Gastric Cancer in an Eastern Chinese Population

**DOI:** 10.1371/journal.pone.0049308

**Published:** 2012-11-15

**Authors:** Jing He, Yu Xu, Li-Xin Qiu, Jin Li, Xiao-Yan Zhou, Meng-Hong Sun, Jiu-Cun Wang, Ya-Jun Yang, Li Jin, Qing-Yi Wei, Yanong Wang

**Affiliations:** 1 Cancer Research Laboratory, Fudan University Shanghai Cancer Center, Shanghai, China; 2 Department of Oncology, Shanghai Medical College, Fudan University, Shanghai, China; 3 Department of Gastric Cancer & Soft Tissue Sarcoma Surgery, Fudan University Shanghai Cancer Center, Shanghai, China; 4 Department of Medical Oncology, Fudan University Shanghai Cancer Center, Shanghai, China; 5 Department of Pathology, Fudan University Shanghai Cancer Center, Shanghai, China; 6 Ministry of Education Key Laboratory of Contemporary Anthropology and State Key Laboratory of Genetic Engineering, School of Life Sciences, Fudan University, Shanghai, China; 7 Fudan-Taizhou Institute of Health Sciences, Taizhou, Jiangsu, China; 8 Department of Epidemiology, The University of Texas, M. D. Anderson Cancer Center, Houston, Texas, United States of America; Huazhong University of Science and Technology, China

## Abstract

**Background:**

Inherited functional single nucleotide polymorphisms (SNPs) in DNA repair genes may alter DNA repair capacity and thus contribute to cancer risk.

**Methods:**

Three *ERCC1* functional SNPs (rs2298881C>A, rs3212986C>A and rs11615G>A) and two *XPF*/*ERCC4* functional SNPs (rs2276466C>G and rs6498486A>C) were genotyped for 1125 gastric adenocarcinoma cases and 1196 cancer-free controls by Taqman assays. Odds ratios (OR) and 95% confidence intervals (CI) were used to estimate risk associations, and false-positive report probabilities (FPRP) were calculated for assessing significant findings.

**Results:**

*ERCC1* rs2298881C and rs11615A variant genotypes were associated with increased gastric cancer risk (adjusted OR = 1.33, 95% CI = 1.05–1.67 for rs2298881 AC/CC and adjusted OR = 1.23, 95% CI = 1.05–1.46 for rs11615 AG/AA, compared with their common genotype AA and GG, respectively). Patients with 2–3 *ERCC1* risk genotypes had significant increased risk (adjusted OR = 1.56, 95% CI = 1.27–1.93), compared with those with 0–1 *ERCC1* risk genotypes, and this risk was more significantly in subgroups of never drinkers, non-gastric cardia adenocarcinoma (NGCA) and clinical stage I+II. All these risks were not observed for *XPF* SNPs.

**Conclusions:**

These findings suggest that functional *ERCC1* SNPs may contribute to risk of gastric cancer. Larger and well-designed studies with different ethnic populations are needed to validate our findings.

## Introduction

Gastric cancer is the second leading cause of cancer-related deaths worldwide, with an estimated nearly one million new cases, accounting for 10% of all cancer-related deaths occurred in 2008 and approximately 40% of all cases occurred in China [1], ranking the third most common cancer in China [2]. To date, the etiology of gastric cancer remains unclear, although currently suggested risk factors include smoking and dietary deficiencies [3], [4], gastroesophageal reflux and obesity [5], high body mass index [6], and *Helicobacter pylori* (*H. pylori*) infection [7]. It is likely that genetic variation may also influence susceptibility to gastric cancer [8].

DNA repair are important in maintaining stability and integrity of human genome. In humans, there are at least five major DNA repair pathways consisting of more than 130 genes, of which the nucleotide excision repair (NER) pathway removes a wide variety of DNA lesions, including bulky adducts, cross links, oxidative DNA damage, alkylating damage and thymidine dimers [9]–[11]. In addition, at least seven xeroderma pigmentosum (XP) complementation groups have been identified, which are the rate-limiting ones in the NER mechanism [12]. For example, the excision repair cross-complimentary group 1 (*ERCC1*) gene encodes a subunit of the NER complex required for the incision step of NER. More importantly, the ERCC1 protein forms a heterodimer with the XPF endonuclease (also known as ERCC4) to catalyze the 5′ incision in the process of excising the DNA lesion [13]. Since the ERCC1 protein is critical for NER and may influence genomic instability, variations in *ERCC1* are likely to play an important role in maintaining genomic stability that is disrupted in carcinogenesis [14], [15].

Three common *ERCC1* variants, namely rs11615, rs321986 and rs3212961, have been investigated previously, because they are probably functional [16]. Specifically, two case-control studies reported the association between *ERCC1* rs11615 variant genotypes and gastric cancer risk, including one study of 126 cases [17] and another of 314 cases [18] in Italian populations. For the association between *ERCC4* polymorphisms and cancer risk, previous studies have mainly focused on a functional SNP rs1800067G>A, and few case-control studies reported on gastric cancer to date [19], [20].

To further assess the associations of *ERCC1* and *ERCC4* polymorphisms with gastric cancer risk, we conducted a case-control study by genotyping five potential functional SNPs, three in *ERCC1* and two in *ERCC4,* in an Eastern Chinese Han population of 1125 gastric cancer cases and 1196 cancer-free controls.

**Table 1:** Logistic regression analysis of associations between selected *ERCC1* and *XPF/ERCC4* SNPs and gastric cancer risk in an Eastern Chinese population.

Variants	Genotypes	Cases(N = 1125)	Controls(N = 1196)	*P* a	Crude OR(95% CI)	*P*	Adjusted OR(95% CI)^b^	*P* b
*ERCC1* rs2298881								
	AA	151 (13.4)	204 (17.1)	**0.037** c	1.00		1.00	
	AC	599 (53.2)	592 (49.5)		**1.37 (1.08–1.74)**	**0.010**	**1.37 (1.08–1.74)**	**0.011**
	CC	375 (33.3)	400 (33.4)		1.27 (0.98–1.63)	0.067	1.26 (0.98–1.63)	0.071
	AC/CC	974 (86.6)	992 (82.9)	**0.015** d	**1.33 (1.06–1.67)**	**0.015**	**1.33 (1.05–1.67)**	**0.016**
*ERCC1* rs3212986								
	CC	526 (46.8)	574 (48.0)	0.814^c^	1.00		1.00	
	AC	489 (43.5)	511 (42.7)		1.04 (0.88–1.24)	0.620	1.03 (0.87–1.23)	0.704
	AA	110 (9.8)	111 (9.3)		1.08 (0.81–1.44)	0.595	1.06 (0.79–1.42)	0.692
	AC/AA	599 (53.2)	622 (52.0)	0.551^d^	1.05 (0.89–1.24)	0.551	1.04 (0.88–1.22)	0.650
*ERCC1* rs11615								
	GG	610 (54.2)	707 (59.1)	**0.0496** c	1.00		1.00	
	AG	454 (40.4)	425 (35.5)		**1.24 (1.04–1.47)**	**0.014**	**1.25 (1.05–1.48)**	**0.011**
	AA	61 (5.4)	64 (5.4)		1.11 (0.77–1.59)	0.596	1.13 (0.78–1.63)	0.521
	AG/AA	515 (45.8)	489 (40.9)	**0.017** d	**1.22 (1.04–1.44)**	**0.018**	**1.23 (1.05–1.46)**	**0.013**
*ERCC4* rs2276466								
	CC	694 (61.7)	735 (61.5)	0.985^c^	1.00		1.00	
	CG	385 (34.2)	413 (34.5)		0.99 (0.83–1.17)	0.885	0.98 (0.83–1.17)	0.853
	GG	46 (4.1)	48 (4.0)		1.02 (0.67–1.54)	0.944	0.99 (0.65–1.51)	0.964
	CG/GG	431 (38.3)	461 (38.5)	0.908^d^	0.99 (0.84–1.17)	0.908	0.98 (0.83–1.17)	0.855
*ERCC4* rs6498486								
	AA	652 (58.0)	694 (58.0)	0.968^c^	1.00		1.00	
	AC	413 (36.7)	441 (36.9)		1.00 (0.84–1.18)	0.971	0.99 (0.83–1.18)	0.917
	CC	60 (5.3)	61 (5.1)		1.05 (0.72–1.52)	0.809	1.05 (0.72–1.52)	0.815
	AC/CC	473 (42.0)	502 (42.0)	0.972^d^	1.00 (0.85–1.18)	0.972	1.00 (0.85–1.18)	0.977
Combined effect of *ERCC1* risk genotypes								
	0	151 (13.4)	197 (16.5)	**<0.0001** c	1.00		1.00	
	1	30 (2.7)	79 (6.6)		**0.50 (0.31–0.79)**	**0.004**	**0.49 (0.31–0.79)**	**0.003**
	2	774 (68.8)	736 (61.5)		**1.37 (1.09–1.74)**	**0.008**	**1.37 (1.08–1.73)**	**0.009**
	3	170 (15.1)	184 (15.4)		1.21 (0.90–1.62)	0.218	1.20 (0.89–1.62)	0.226
						
	0–1	181 (16.1)	276 (23.1)	**<0.0001**	1.00		1.00	
	2–3	944 (83.9)	920 (76.9)		**1.57 (1.27–1.93)**	**<0.0001**	**1.56 (1.27–1.93)**	**<0.0001**

SNP, single-nucleotide polymorphism; CI, confidence interval; OR, odds ratio.

The results were in bold, if the 95% CI excluded 1 or *P*<0.05.

a Chi square test for genotype distributions between cases and controls.

b Adjusted for age, sex, smoking and drinking status in logistic regress models.

c For additive genetic models.

d For dominant genetic models.

## Materials and Methods

### Study Population

The subjects were recruited from an ongoing case-control study as described previously [21]. This study included 1125 unrelated ethnic Han Chinese patients with newly diagnosed and histopathologically confirmed primary gastric adenocarcinoma recruited from Fudan University Shanghai Cancer Center (FUSCC) between January 2009 and March 2011. All the patients were from Eastern China, including Shanghai, Zhejiang, Jiangsu and the surrounding regions. Because gastric adenocarcinoma accounts for nearly 98.5% of all gastric cancer patients seen at our hospital, we excluded from participation those patients who had interstitialoma, gastric adenosquamous carcinoma, squamous cell carcinoma, metastasized cancer from other organs or any histopathologic diagnosis other than gastric adenocarcinoma. An additional 1196 controls of age- (±5 years) and sex-matched cancer-free ethnic Han Chinese with written informed consent were recruited from the Taizhou Longitudinal (TZL) study conducted at the same time period in Eastern China which was approved by the Human Ethics Committee of Fudan University as described previously [22]. All blood samples from patients with gastric adenocarcinoma were provided by the tissue bank of FUSCC. All patients had signed a written informed consent for donating their biological samples to the tissue bank of FUSCC. The response rate was approximately 91% for cases and 90% for controls. This research protocol was approved by the Institutional Review Board of FUSCC.

### SNP Selection and Genotyping

We selected potentially functional SNPs of interest by using the NCBI dbSNP database and SNPinfo with the following criteria: (1) the minor allele frequency reported in HapMap was ≥5% for Chinese subjects; (2) affecting transcription factor binding site (TFBS) activity in the putative promoter region; (3) affecting the microRNA (miRNA) binding site activity; and (4) not included in the published GWASs. For the *ERCC1* gene, we chose rs2298881C>A that may affect the TFBS activity, in addition to other two widely investigated potentially functional SNPs (rs3212986C>A and rs11615G>A, the former may be associated with transcript stability alterations, while the latter may be associated with mRNA levels alterations). For the *ERCC4* gene, we chose rs2276466C>G and rs6498486A>C; the former may affect the miRNA binding site activity, while the latter may affect the TFBS activity as predicted by the SNPinfo. These five potentially functional SNPs also captured other 49 SNPs in the nearby genes (**Table S1** for *ERCC1* and **Table S2** for *ERCC4*) and were genotyped as described previously [21].

**Table 2:** Stratification analysis for associations between *ERCC1* variant genotypes and gastric cancer risk in an Eastern Chinese population.

Variables	rs2298881 (cases/controls)		Adjusted OR^a^ (95% CI)	*P* a	*P* ^het^	rs3212986 (cases/controls)		Adjusted OR^a^ (95% CI)	*P* a	*P* ^het^	rs11615 (cases/controls)		Adjusted OR^a^ (95% CI)	*P* a	*P^het^*	Combined effect of risk genotypes (cases/controls)		Adjusted OR^a^ (95% CI)	*P* a	*P* ^het^
	AA	AC/CC				CC	AC/AA				GG	AG/AA				0–1	2–3			
Median age, yr																				
≤59	79/107	499/503	1.35 (0.98–1.85)	0.064	0.910	277/288	301/322	0.98 (0.78–1.23)	0.850	0.335	308/369	270/241	**1.34 (1.07–1.69)**	**0.013**	0.248	96/144	482/466	**1.55 (1.16–2.07)**	**0.003**	0.931
>59	72/97	475/489	1.35 (0.97–1.89)	0.080		249/286	298/300	1.13 (0.89–1.44)	0.306		302/338	245/248	1.14 (0.90–1.45)	0.283		85/132	462/454	**1.62 (1.19–2.20)**	**0.002**	
Sex																				
Male	115/141	685/685	1.23 (0.94–1.61)	0.135	0.261	385/406	415/420	1.03 (0.85–1.25)	0.783	0.823	439/484	361/342	1.18 (0.97–1.44)	0.102	0.382	136/192	664/634	**1.48 (1.16–1.89)**	**0.002**	0.374
Female	36/63	289/307	**1.64 (1.05–2.56)**	**0.029**		141/168	184/202	1.05 (0.77–1.42)	0.767		171/223	154/147	**1.38 (1.02–1.88)**	**0.036**		45/84	280/286	**1.82 (1.22–2.72)**	**0.003**	
Smoking status																				
Never	92/99	594/511	1.27 (0.93–1.74)	0.128	0.704	307/281	379/329	1.08 (0.87–1.35)	0.502	0.688	386/365	300/245	1.15 (0.92–1.44)	0.210	0.622	110/135	576/475	**1.50 (1.13–1.99)**	**0.005**	0.405
Former	3/18	14/102	0.73 (0.18–2.89)	0.652		9/52	8/68	0.62 (0.21–1.80)	0.378		8/68	9/52	1.60 (0.55–4.63)	0.388		4/24	13/96	0.72 (0.21–2.48)	0.606	
Current	56/87	366/379	**1.49 (1.03–2.14)**	**0.035**		210/241	212/225	1.05 (0.81–1.36)	0.745		216/274	206/192	**1.38 (1.06–1.80)**	**0.018**		67/117	355/349	**1.76 (1.26–2.47)**	**0.001**	
Drinking status																				
Never	114/142	741/709	**1.31 (1.00–1.71)**	**0.0497**	0.826	383/404	472/447	1.12 (0.92–1.35)	0.260	0.178	475/492	380/359	1.10 (0.91–1.33)	0.337	0.032	138/191	717/660	**1.51 (1.18–1.93)**	**0.001**	0.569
Ever	37/62	233/283	1.41 (0.90–2.19)	0.135		143/170	127/175	0.87 (0.63–1.20)	0.404		135/215	135/130	**1.67 (1.20–2.31)**	**0.002**		43/85	227/260	**1.75 (1.16–2.64)**	**0.007**	
Tumor site																				
GCA	42/204	263/992	1.31 (0.91–1.88)	0.150	0.855	158/574	147/622	0.87 (0.67–1.12)	0.268	0.078	164/707	141/489	1.24 (0.96–1.60)	0.106	0.875	52/276	253/920	**1.48 (1.07–2.07)**	**0.020**	0.638
NGCA	109/204	711/992	**1.33 (1.03–1.71)**	**0.027**		368/574	452/622	1.11 (0.93–1.33)	0.250		446/707	374/489	**1.23 (1.03–1.47)**	**0.026**		129/276	691/920	**1.59 (1.26–2.01)**	**<0.0001**	
Clinical stage																				
I+II	54/204	422/992	**1.58 (1.15–2.18)**	**0.005**	0.134	221/574	255/622	1.05 (0.85–1.30)	0.652	0.876	248/707	228/489	**1.33 (1.08–1.65)**	**0.009**	0.313	68/276	408/920	**1.78 (1.33–2.38)**	**0.0001**	0.223
III+IV	97/204	552/992	1.17 (0.90–1.53)	0.237		305/574	344/622	1.03 (0.85–1.25)	0.771		362/707	287/489	1.15 (0.95–1.40)	0.158		113/276	536/920	**1.42 (1.11–1.81)**	**0.005**	

GCA, gastric cardia adenocarcinoma; NGCA, non-gastric cardia adenocarcinoma.

The results were in bold, if the 95% CI excluded 1 or *P*<0.05.

a Obtained in logistic regression models with adjustment for age, sex, smoking and drinking status.

**Table 3:** False-positive report probability values for associations between the risk of gastric cancer and the frequency of genotypes of the *ERCC1* gene.

Genotype	Crude OR (95% CI)	*P* a	Statistical Power^b^	Prior probability				
				0.25	0.1	0.01	0.001	0.0001
*ERCC1* rs2298881								
AC vs. AA	1.37 (1.08–1.74)	0.010	0.989	0.031	0.086	0.510	0.913	0.991
AC/CC vs. AA	1.33 (1.06–1.67)	0.015	0.838	0.052	0.141	0.644	0.948	0.995
AC/CC vs. AA								
Female	1.65 (1.06–2.56)	0.026	0.354	0.181	0.399	0.879	0.987	0.999
Current smoker	1.50 (1.04–2.16)	0.030	0.503	0.150	0.345	0.853	0.983	0.998
NGCA	1.34 (1.04–1.73)	0.022	0.795	0.077	0.201	0.734	0.965	0.996
Stage I+II	1.61 (1.17–2.21)	0.004	0.353	0.031	0.088	0.516	0.915	0.991
*ERCC1* rs11615								
AG vs. GG	1.24 (1.04–1.47)	0.014	0.990	0.042	0.116	0.590	0.936	0.993
AG/AA vs. GG	1.22 (1.04–1.44)	0.018	0.994	0.050	0.137	0.636	0.946	0.994
AG/AA vs. GG								
≤59	1.34 (1.07–1.69)	0.012	0.832	0.042	0.117	0.594	0.937	0.993
Female	1.37 (1.01–1.85)	0.042	0.731	0.148	0.343	0.852	0.983	0.998
Current smoker	1.36 (1.04–1.78)	0.023	0.766	0.082	0.212	0.747	0.968	0.997
Ever drink	1.65 (1.20–2.28)	0.002	0.279	0.024	0.069	0.449	0.892	0.988
NGCA	1.21 (1.01–1.45)	0.035	0.991	0.097	0.243	0.779	0.973	0.997
Stage I+II	1.33 (1.07–1.65)	0.009	0.868	0.030	0.085	0.507	0.912	0.990
*ERCC1* risk genotypes								
1 vs. 0	0.50 (0.31–0.79)	0.004	0.228	0.044	0.121	0.603	0.939	0.994
2 vs. 0	1.37 (1.09–1.74)	0.008	0.976	0.025	0.071	0.457	0.895	0.988
2–3 vs. 0–1	1.57 (1.27–1.93)	<0.0001	0.361	0	0.001	**0.007**	0.065	0.409
2–3 vs. 0–1								
≤59	1.55 (1.16–2.07)	0.003	0.420	0.020	0.057	0.398	0.870	0.985
>59	1.58 (1.17–2.14)	0.003	0.382	0.023	0.066	0.437	0.887	0.987
Male	1.48 (1.16–1.89)	0.002	0.549	0.010	0.029	0.245	0.766	0.970
Female	1.83 (1.23–2.72)	0.003	0.188	0.046	0.126	0.613	0.941	0.994
Never smoker	1.49 (1.13–1.97)	0.005	0.524	0.029	0.083	0.500	0.910	0.990
Current smoker	1.78 (1.27–2.48)	0.001	0.185	0.013	0.038	0.300	0.812	0.977
Never drinker	1.50 (1.18–1.92)	0.001	0.496	0.006	0.018	**0.166**	0.668	0.953
Ever drinker	1.73 (1.15–2.59)	0.009	0.267	0.089	0.226	0.763	0.970	0.997
GCA	1.46 (1.05–2.02)	0.024	0.567	0.111	0.272	0.805	0.976	0.998
NGCA	1.61 (1.28–2.03)	<0.0001	0.298	0.001	0.002	**0.019**	0.163	0.661
Stage I+II	1.80 (1.35–2.41)	<0.0001	0.124	0.002	0.005	**0.053**	0.361	0.850
Stage III+IV	1.42 (1.12–1.82)	0.005	0.660	0.020	0.058	0.403	0.872	0.986

a Chi-square test was used to calculate the genotype frequency distributions.

b Statistical power was calculated using the number of observations in the subgroup and the OR and *P* values in this table.

### Statistical Analysis

We used χ^2^ test to compare the differences in the frequencies of alleles and genotypes as well as demographic and other covariates between the cases and controls. The goodness-of-fit χ^2^ test was employed to calculate the Hardy–Weinberg equilibrium of genotype distributions in the controls. We calculated crude and adjusted odds ratios (ORs) and 95% confidence intervals (CIs) by univariate and multivariate logistic regression models, respectively, to evaluate associations between the genotypes and risk of gastric adenocarcinoma with and without adjustment for and stratified by age, sex, smoking/drinking status, primary tumor site and clinical stage. We also performed homogeneity tests to detect the difference in risk estimates among subgroups. Because the selected SNPs appear to be in the same block, the haplotype analysis was not performed.

We calculated the false-positive report probability (FPRP) [23] for all significant findings. We set 0.2 as an FPRP threshold and assigned a prior probability of 0.01 to detect an OR of 1.50 (for risk effects) or 0.67 (for protective effects) for an association with genotypes under investigation. Only significant results with an FPRP value <0.2 were considered as noteworthy associations.

We performed all analyses with SAS software (version 9.1; SAS Institute, Cary, NC), and all statistical tests were two-sided, and *P* values less than 0.05 were considered statistically significant.

## Results

### Characteristics of the Patients

The distributions of demographic characteristics of the subjects are presented in **Table S3**, and were similar to those presented in a previous report [21]. Briefly, the current case-control study included 1196 cancer-free controls and 1125 gastric cancer cases, including 305 (27.1%) gastric cardia adenocarcinoma (GCA) and 820 (72.9%) NGCA, of which 476 (42.3%) were of TNM stage I+II, and 649 (57.7%) were of stage III+IV according to the 7^th^ Edition of the AJCC [24].

### Association between Selected SNPs and Gastric Cancer Risk

The genotype distributions of the five selected SNPs in cases and controls are shown in **Table 1**. The observed genotype distributions among the controls were agreed with the Hardy–Weinberg equilibrium (*P* = 0.550 for rs2298881, *P* = 0.859 for rs3212986, *P* = 0.990 for rs11615, *P* = 0.288 for rs2276466, and *P* = 0.398 for rs6498486). The genotype distributions were significantly different for *ERCC1* rs2298881 (*P* = 0.037) and *ERCC1* rs11615 (*P* = 0.0496) between the cases and controls, but not for the other three SNPs. When the rs2298881AA genotype was used as the reference, the C variant genotypes were associated with an increased risk of gastric adenocarcinoma (adjusted OR = 1.37, 95% CI = 1.08–1.74 for AC, adjusted OR = 1.26, 95% CI = 0.98–1.63 for CC and adjusted OR = 1.33; 95% CI = 1.05–1.67 for AC/CC after adjustment for age, sex, smoking and drinking status); when the rs11615GG genotype was used as the reference, the A variant genotypes were associated with an increased risk of gastric adenocarcinoma (adjusted OR = 1.25, 95% CI = 1.05–1.48 for AG, adjusted OR = 1.13, 95% CI = 0.78–1.63 for AA and adjusted OR = 1.23; 95% CI = 1.05–1.46 for AG/AA). Nevertheless, no associations with risk of gastric adenocarcinoma were found for other three SNPs (**Table 1**). Patients with 2–3 risk genotypes of *ERCC1* had significant increased risk (adjusted OR = 1.56, 95% CI = 1.27–1.93), compared with those with only 0–1 risk genotypes.

### Stratification Analysis

We further evaluated the association between variant genotypes of three selected SNPs of *ERCC1* and gastric cancer risk stratified by subgroups of age, sex, smoking and drinking status, tumor site and clinical stage, assuming a dominant genetic model based on the results from univariate analysis (**Table 2**). The rs2298881 variant AC/CC genotypes was associated with an increased risk (adjusted ORs unless otherwise specified) that was more evident in females (OR = 1.64, 95% CI = 1.05–2.56), current-smokers (OR = 1.49, 95% CI = 1.03–2.14), never drinkers (OR = 1.31, 95% CI = 1.00–1.71), NGCA (OR = 1.33, 95% CI = 1.03–1.71) and clinical stage I+II (OR = 1.58, 95% CI = 1.15–2.18). Quite similar results were found for rs11615 variant AG/AA genotypes, especially in the younger group (OR = 1.34, 95% CI = 1.07–1.69), females (OR = 1.38, 95% CI = 1.02–1.88), current-smokers (OR = 1.38, 95% CI = 1.06–1.80), ever drinkers (OR = 1.67, 95% CI = 1.20–2.31), NGCA (OR = 1.23, 95% CI = 1.03–1.47) and clinical stage I+II (OR = 1.33, 95% CI = 1.08–1.65). When all risk genotypes were combined into a new variable, we found that the patients carrying 2–3 risk genotypes had a more evident risk in older group (OR = 1.62, 95% CI = 1.19–2.20), females (OR = 1.82, 95% CI = 1.22–2.72), current-smokers (OR = 1.76, 95% CI = 1.26–2.47), ever drinkers (OR = 1.75, 95% CI = 1.16–2.64), NGCA (OR = 1.59, 95% CI = 1.26–2.01) and clinical stage I+II (OR = 1.78, 95% CI = 1.33–2.38). However, further homogeneity tests suggested no differences in the risk estimates among the strata without statistical evidence of interactions between these variables and the variant genotypes on the risk (data not shown).

The FPRP values at different prior probability levels for significant findings are shown in **Table 3**. For a prior probability of 0.01, assuming that the OR for specific genotype was 1.50 (risk) or 0.67 (protection), with statistical power of 0.361, the FPRP values were 0.007 for an association of the *ERCC1* 2–3 risk genotypes, with an increased risk of gastric adenocarcinoma in all individuals. We also found a significant association with gastric adenocarcinoma risk from never drinkers, NGCA and clinical stage I+II among those patients with 2–3 risk genotypes. These significant associations were considered as noteworthy, because the probability of a false-positive result was <20%. In contrast, greater FPRP values were observed for other significant associations between *ERCC*1 variants and gastric adenocarcinoma, suggesting some possible bias in these positive findings.

## Discussion

In this study, we found that *ERCC1* rs2298881C and rs11615A variant genotypes were associated with a pronounced increased risk of gastric adenocarcinoma and that the increased risk associated with 2–3 risk genotypes was more evident in never drinkers, NGCA and clinical stage I+II. To our knowledge, this is the first study that *ERCC1* intron rs2298881 C/A and coding region rs11615 G/A polymorphisms were found to be associated with gastric adenocarcinoma risk. Because ERCC1 plays a critical role in NER, these findings are biologically plausible.

*ERCC1* has been mapped to chromosome 19q13.32, consisting of 10 exons and encoding a 297 acetaldehyde ammonia product, while *ERCC4,* located on 16p13.12, comprises 11 exons and encodes a 916 acetaldehyde ammonia product. *ERCC1* encodes a subunit for the NER complex that was required for the incision step of the NER pathway [15], [25]. Importantly, a heterodimer of ERCC1/XPF catalyzes the 5′ incision in the process of excising DNA lesions in recombinational DNA repair and in the repair of inter-strand crosslinks [26]–[28]. Thus, it is possible that functional *ERCC1* variants may also play a role in cancer risk. For example, the *ERCC1* Asn118Asn (rs11615) SNP has been reported to be associated with some specific subtype of lung cancer as well as early onset of lung cancer [29]. Likewise, the *ERCC1* 19007 C (rs11615) allele was found to be associated with an elevated lung cancer risk in Asian populations [16]. To date, in the published studies on associations between the *ERCC1* rs11615T>C, rs3212986C>A and rs3212961A>C polymorphisms and cancer risk [16], only two studies focused on *ERCC1* polymorphisms and risk of gastric cancer, both of which studied the rs11615T>C only in a relatively small Italy population, including one [17] with only 126 gastric cancer cases and the other of only 314 cases [18], but none of these two studies included the SNPs under investigation in the present study.

When we combined these three selected *ERCC1* SNPs, we found that patients with 2–3 risk genotypes had significantly increased risk of gastric cancer compared with those with 0–1 risk genotypes. In the stratified analysis, we also found that the effect of the rs2298881 AC/CC genotypes on cancer risk was more evident in subgroups of females, current-smokers, never drinkers, NGCA and clinical stage I+II and that quite similar results were for rs11615 variant AG/AA genotypes, especially in younger subjects, females, current-smokers, ever drinkers, NGCA and clinical stage I+II. However, after FPRP was calculated for these significant findings, only the findings for never drinkers, NGCA and clinical stage I+II remained significant for the patients with 2–3 risk genotypes. Therefore, some of our findings from the stratified analysis may be chance findings because of reduced sample sizes in the subgroups. For example, we found that the ever-drinkers had a more evident risk (OR = 1.75, 95% CI = 1.16–2.64) than the never-drinkers (OR = 1.51, 95% CI = 1.18–1.93); however, based on the FPRP calculation, the risk found for the ever-drinkers was not significant for a prior probability of 0.01. This may be ascribed to the reduction of sample sizes, for there were more never-drinkers than ever-drinkers in our current study. Tobacco smoke-related carcinogens may induce various kinds of DNA damage that mainly removed by the NER pathway [30]. If unrepaired, such DNA damage may lead to mutations and thus the initiation of carcinogenesis. For example, DNA adducts identified in fetal blood may increase subsequent risk of developing cancer in the adulthood [31]. For current smokers, the effect of genetic instability on cancer risk may be augmented by accumulated DNA damage caused by continued cigarette smoking. Females may be more susceptible to second-hand smoke or affected by indoor air pollution from unventilated coal-fueled stoves and from cooking fumes [32]. Gastric cancer includes NGCA and GCA, and GCA is localized to the gastroesophageal junction and differs from NGCA in epidemiological characteristics and clinical features [4], [33]. Finally, the finding that patients with 2–3 *ERCC1* risk genotypes had increased risk of having early-stage cancer may suggest that the observed risk could be genetic susceptibility as the cause for carcinogenesis of the target tissue rather than tumor progression that could be driven by additional mutational events [34].

In summary, this large hospital-based case-control study provided statistical evidence that *ERCC1* rs2298881 and rs11615 SNPs, but not *ERCC4* SNPs, were associated with gastric cancer risk in an Eastern Chinese population, particularly for never-drinkers, patients with NGCA and early clinical stages. However, the present study had several limitations. First, the patients were selected from FUSCC that did not have a well-defined catchment area for the cases in Eastern China as the TZL study for the controls, which may have selection bias and information bias. Second, only three potential functional SNPs of *ERCC1* and two of *ERCC4* were investigated in the present study, which did not cover all SNPs of the *ERCC1/XPF* complex. Finally, we did not have reliable and sufficient information on other environmental exposures, such as dietary intake, occupation and *H. pylori* infection, due to the nature of the retrospective study design. Because our patients were mainly from Eastern China, it would be ideal to have a multi-center based replication to validate our findings. Without such replication, our findings should be considered preliminary. With such preliminary findings from the present study, we hope more cancer research centers or laboratories in China and other regions of the world with a high incidence of gastric cancer to validate our findings with larger sample sizes, more complete information on dietary intake, occupation and H. pylori infection.

## Supporting Information

Table S1 SNPs captured by the selected three *ERCC1* functional SNPs as predicted by SNPinfo software.
(DOC)

Table S2 SNPs captured by the selected two *ERCC4* functional SNPs as predicted by SNPinfo software.
(DOC)

Table S3 Frequency distribution of demographic characteristics of gastric cancer cases and cancer-free controls.
(DOC)
