# Supplementary material for: Polymorphisms in ERCC1 and XPF Genes and Risk of Gastric Cancer in an Eastern Chinese Population
Source: PLoS One. 2012 Nov 15;7(11):e49308. doi: 10.1371/journal.pone.0049308 (PMC3499547; doi:10.1371/journal.pone.0049308)
Supplement: Table S1 — SNPs captured by the selected three ERCC1 functional SNPs as predicted by SNPinfo software. (DOC) [file pone.0049308.s001.doc]

**Table S1.** SNPs captured by the selected three *ERCC1* functional SNPs as predicted by SNPinfo software.

| **rs** | **Chr** | **Allele** | **LDsnp** | **Pop/LD** | **TFBS** | **Splicing (ESE or ESS)** | **miRNA (miRanda)** | **miRNA (Sanger)** | **nsSNP** | **Polyphen** | **Reg Potential** | **Conservation** | **Nearby Gene** | **Distance (bp)** | **Allele** | **Asian** | **CHB** |
| --- | --- | --- | --- | --- | --- | --- | --- | --- | --- | --- | --- | --- | --- | --- | --- | --- | --- |
| rs10412761 | 19 | A/G | rs11615 | CHB/0.841 | Y | -- | -- | -- | -- | -- | 0.269614 | 0 | PPP1R13L||CD3EAP | -172||-1006 | G | 0.772 | 0.750 |
| rs11615 | 19 | G/A | rs11615 | 1 | -- | Y | -- | -- | -- | -- | 0.26724 | 0.989 | ERCC1 | 10781||3524 | G | 0.754 | 0.750 |
| rs11882642 | 19 | C/T | rs11615 | CHB/0.969 | -- | -- | -- | -- | -- | -- | 0 | 0.293 | ERCC1||FOSB | -6316||-37760 | C | 0.756 | 0.741 |
| rs1319052 | 19 | A/G | rs11615 | CHB/1.000 | Y | -- | -- | -- | -- | -- | 0.155417 | 0.001 | ERCC1||FOSB | -4044||-40032 | A | 0.758 | 0.778 |
| rs3212948 | 19 | G/C | rs11615 | CHB/1.000 | -- | -- | -- | -- | -- | -- | 0 | 0 | ERCC1 | 11490||2815 | C | 0.742 | 0.778 |
| rs7248764 | 19 | C/T | rs11615 | CHB/1.000 | -- | -- | -- | -- | -- | -- | NA | 0 | ERCC1||FOSB | -7830||-36246 | C | 0.758 | 0.778 |
| rs2298881 | 19 | A/C | rs2298881 | 1 | Y | -- | -- | -- | -- | -- | 0.252611 | 0 | ERCC1 | 14044||261 | C | 0.522 | 0.566 |
| rs1046282 | 19 | G/A | rs3212986 | CHB/0.973 | Y | -- | -- | -- | -- | -- | NA | 0 | CD3EAP | 1205||3352 | A | -- | 0.673 |
| rs3212965 | 19 | G/A | rs3212986 | CHB/0.947 | -- | -- | -- | -- | -- | -- | 0 | 0.007 | ERCC1 | 7708||6597 | G | 0.757 | 0.689 |
| rs3212980 | 19 | T/G | rs3212986 | CHB/1.000 | -- | -- | -- | -- | -- | -- | NA | 0 | ERCC1 | 3569||10736 | T | -- | 0.679 |
| rs3212986 | 19 | C/A | rs3212986 | 1 | -- | -- | -- | -- | Y | benign | 0.305187 | 0 | CD3EAP | 3269||1288 | C | 0.761 | 0.679 |
| rs4803817 | 19 | A/G | rs3212986 | CHB/0.973 | Y | -- | -- | -- | -- | -- | 0.073744 | 0 | PPP1R13L | 25064||329 | A | 0.756 | 0.673 |
