# Supplementary material for: Polymorphisms in ERCC1 and XPF Genes and Risk of Gastric Cancer in an Eastern Chinese Population
Source: PLoS One. 2012 Nov 15;7(11):e49308. doi: 10.1371/journal.pone.0049308 (PMC3499547; doi:10.1371/journal.pone.0049308)
Supplement: Table S2 — SNPs captured by the selected two ERCC4 functional SNPs as predicted by SNPinfo software. (DOC) [file pone.0049308.s002.doc]

**Table S2.** SNPs captured by the selected two *ERCC4* functional SNPs as predicted by SNPinfo software.

| **rs** | **Chr** | **Allele** | **LDsnp** | **Pop/LD** | **TFBS** | **Splicing (ESE or ESS)** | **miRNA (miRanda)** | **miRNA (Sanger)** | **Reg Potential** | **Conservation** | **Nearby Gene** | **Distance (bp)** | **Allele** | **Asian** | **CHB** |
| --- | --- | --- | --- | --- | --- | --- | --- | --- | --- | --- | --- | --- | --- | --- | --- |
| rs2276466 | 16 | C/G | rs2276466|rs6498486 | 1|CHB/0.935 | -- | -- | Y | Y | 0.174235 | 0 | ERCC4||MKL2 | -971||-122021 | C | 0.73 | 0.784 |
| rs12921111 | 16 | A/G | rs6498486 | CHB/0.930 | Y | -- | -- | -- | 0 | 0 | LOC729993||ERCC4 | -680557||-4200 | G | 0.736 | 0.795 |
| rs7197700 | 16 | A/G | rs6498486 | CHB/0.929 | -- | -- | -- | -- | NA | 0.001 | LOC729993||ERCC4 | -673706||-11051 | G | 0.735 | 0.786 |
| rs7200442 | 16 | C/T | rs6498486 | CHB/0.863 | -- | -- | -- | -- | NA | 0 | LOC729993||ERCC4 | -674390||-10367 | C | 0.728 | 0.772 |
| rs11075223 | 16 | A/C | rs6498486|rs2276466 | CHB/0.902|CHB/1.000 | -- | -- | Y | -- | 0 | 0 | ERCC4||MKL2 | -2513||-120479 | C | 0.744 | 0.786 |
| rs11639593 | 16 | C/G | rs6498486|rs2276466 | CHB/0.935|CHB/0.809 | Y | -- | -- | -- | 0.128221 | 0.699 | LOC729993||ERCC4 | -680210||-4547 | C | 0.725 | 0.773 |
| rs11648436 | 16 | C/T | rs6498486|rs2276466 | CHB/0.934|CHB/0.867 | -- | -- | -- | -- | 0 | 0 | LOC729993||ERCC4 | -679408||-5349 | C | 0.728 | 0.768 |
| rs11648736 | 16 | A/G | rs6498486|rs2276466 | CHB/0.873|CHB/0.934 | -- | -- | -- | -- | 0.157599 | 0.011 | ERCC4||MKL2 | -8688||-114304 | G | 0.754 | 0.8 |
| rs11649492 | 16 | C/G | rs6498486|rs2276466 | CHB/0.936|CHB/0.810 | Y | -- | -- | -- | 0 | 0 | LOC729993||ERCC4 | -680152||-4605 | G | 0.736 | 0.778 |
| rs12924220 | 16 | C/T | rs6498486|rs2276466 | CHB/0.933|CHB/0.865 | -- | -- | -- | -- | NA | 0.002 | LOC729993||ERCC4 | -675867||-8890 | C | 0.728 | 0.762 |
| rs12926685 | 16 | C/T | rs6498486|rs2276466 | CHB/0.934|CHB/0.867 | Y | -- | -- | -- | 0 | 0 | LOC729993||ERCC4 | -680553||-4204 | T | 0.725 | 0.768 |
| rs1646332 | 16 | C/A | rs6498486|rs2276466 | CHB/0.901|CHB/1.000 | -- | -- | -- | -- | 0 | 0.003 | ERCC4||MKL2 | -7523||-115469 | C | 0.75 | 0.774 |
| rs1799797 | 16 | A/T | rs6498486|rs2276466 | CHB/1.000|CHB/0.878 | Y | -- | -- | -- | 0.288343 | 0 | LOC729993||ERCC4 | -684727||-30 | T | 0.73 | 0.767 |
| rs1799800 | 16 | A/G | rs6498486|rs2276466 | CHB/0.934|CHB/1.000 | -- | -- | -- | -- | 0 | 0 | ERCC4 | 24529||3652 | G | 0.742 | 0.78 |
| rs1799801 | 16 | C/T | rs6498486|rs2276466 | CHB/0.868|CHB/1.000 | -- | -- | -- | -- | 0.205381 | 0.326 | ERCC4 | 27935||246 | T | 0.757 | 0.78 |
| rs2238462 | 16 | C/G | rs6498486|rs2276466 | CHB/0.933|CHB/0.812 | -- | -- | -- | -- | 0.16461 | 0 | ERCC4 | 1287||26894 | C | 0.729 | 0.767 |
| rs2276465 | 16 | A/G | rs6498486|rs2276466 | CHB/0.936|CHB/1.000 | -- | -- | Y | -- | 0.13319 | 0 | ERCC4||MKL2 | -810||-122182 | G | 0.754 | 0.789 |
| rs3136038 | 16 | C/T | rs6498486|rs2276466 | CHB/1.000|CHB/0.935 | Y | -- | -- | -- | 0.067995 | 0 | LOC729993||ERCC4 | -684113||-644 | C | 0.75 | 0.765 |
| rs3136079 | 16 | G/T | rs6498486|rs2276466 | CHB/0.936|CHB/1.000 | -- | -- | -- | -- | 0 | 0 | ERCC4 | 5214||22967 | T | 0.761 | 0.789 |
| rs3136130 | 16 | G/T | rs6498486|rs2276466 | CHB/0.936|CHB/1.000 | -- | -- | -- | -- | NA | 0 | ERCC4 | 12928||15253 | G | 0.733 | 0.789 |
| rs3136155 | 16 | C/T | rs6498486|rs2276466 | CHB/0.936|CHB/1.000 | -- | -- | -- | -- | 0 | 0 | ERCC4 | 16337||11844 | C | 0.759 | 0.789 |
| rs3136166 | 16 | G/T | rs6498486|rs2276466 | CHB/0.902|CHB/1.000 | -- | -- | -- | -- | 0 | 0.001 | ERCC4 | 18070||10111 | T | 0.733 | 0.786 |
| rs3136168 | 16 | G/T | rs6498486|rs2276466 | CHB/0.933|CHB/1.000 | -- | -- | -- | -- | 0.136461 | 0 | ERCC4 | 18431||9750 | T | 0.69 | 0.756 |
| rs3136173 | 16 | G/T | rs6498486|rs2276466 | CHB/0.936|CHB/1.000 | -- | -- | -- | -- | 0 | 0.001 | ERCC4 | 19044||9137 | G | 0.733 | 0.789 |
| rs3136176 | 16 | A/T | rs6498486|rs2276466 | CHB/0.936|CHB/1.000 | -- | -- | -- | -- | 0 | 0.002 | ERCC4 | 19273||8908 | A | 0.754 | 0.789 |
| rs3136187 | 16 | A/G | rs6498486|rs2276466 | CHB/0.934|CHB/1.000 | -- | -- | -- | -- | 0.050665 | 0 | ERCC4 | 20476||7705 | A | 0.742 | 0.78 |
| rs3136189 | 16 | C/T | rs6498486|rs2276466 | CHB/0.934|CHB/1.000 | -- | -- | -- | -- | 0.139222 | 0.005 | ERCC4 | 20678||7503 | T | 0.733 | 0.78 |
| rs3136202 | 16 | A/G | rs6498486|rs2276466 | CHB/0.934|CHB/1.000 | -- | -- | -- | -- | 0 | 0 | ERCC4 | 24151||4030 | G | 0.75 | 0.777 |
| rs3136211 | 16 | C/G | rs6498486|rs2276466 | CHB/0.934|CHB/1.000 | -- | -- | -- | -- | 0.049693 | 0 | ERCC4 | 25625||2556 | C | 0.728 | 0.78 |
| rs3136218 | 16 | G/T | rs6498486|rs2276466 | CHB/0.936|CHB/1.000 | -- | -- | -- | -- | NA | 0 | ERCC4 | 26575||1606 | T | 0.733 | 0.789 |
| rs3743538 | 16 | G/T | rs6498486|rs2276466 | CHB/0.902|CHB/1.000 | -- | -- | Y | Y | 0 | 0 | ERCC4||MKL2 | -484||-122508 | G | 0.759 | 0.786 |
| rs3784872 | 16 | C/T | rs6498486|rs2276466 | CHB/0.936|CHB/1.000 | -- | -- | -- | -- | 0 | 0 | ERCC4||MKL2 | -4060||-118932 | T | 0.744 | 0.789 |
| rs4781560 | 16 | C/T | rs6498486|rs2276466 | CHB/0.936|CHB/0.810 | -- | -- | -- | -- | NA | 0.003 | LOC729993||ERCC4 | -676485||-8272 | T | 0.746 | 0.778 |
| rs4781562 | 16 | G/T | rs6498486|rs2276466 | CHB/0.902|CHB/1.000 | -- | -- | Y | -- | 0 | 0 | ERCC4||MKL2 | -3032||-119960 | G | 0.833 | 0.786 |
| rs4781563 | 16 | A/G | rs6498486|rs2276466 | CHB/0.902|CHB/1.000 | -- | -- | Y | -- | 0 | 0 | ERCC4||MKL2 | -3195||-119797 | G | 0.754 | 0.786 |
| rs6498485 | 16 | A/G | rs6498486|rs2276466 | CHB/0.936|CHB/0.810 | Y | -- | -- | -- | 0.063774 | 0 | LOC729993||ERCC4 | -681728||-3029 | G | 0.736 | 0.778 |
| rs6498486 | 16 | A/C | rs6498486|rs2276466 | 1|CHB/0.935 | Y | -- | -- | -- | 0.174037 | 0 | LOC729993||ERCC4 | -684400||-357 | A | 0.746 | 0.768 |
| rs7186212 | 16 | C/T | rs6498486|rs2276466 | CHB/0.934|CHB/0.867 | -- | -- | -- | -- | NA | 0 | LOC729993||ERCC4 | -679531||-5226 | T | 0.728 | 0.768 |
| rs744154 | 16 | G/C | rs6498486|rs2276466 | CHB/1.000|CHB/0.873 | -- | -- | -- | -- | 0 | 0.001 | ERCC4 | 1058||27123 | G | 0.697 | 0.773 |
| rs889817 | 16 | G/A | rs6498486|rs2276466 | CHB/0.936|CHB/0.810 | -- | -- | -- | -- | 0 | 0 | LOC729993||ERCC4 | -670935||-13822 | A | 0.728 | 0.778 |
| rs9646271 | 16 | C/T | rs6498486|rs2276466 | CHB/0.902|CHB/1.000 | -- | -- | -- | -- | 0 | 0 | ERCC4||MKL2 | -5214||-117778 | C | 0.754 | 0.786 |
| rs9646272 | 16 | C/G | rs6498486|rs2276466 | CHB/0.936|CHB/1.000 | -- | -- | -- | -- | 0 | 0 | ERCC4||MKL2 | -6640||-116352 | G | 0.75 | 0.789 |
