# Supplementary material for: Polymorphisms in ERCC1 and XPF Genes and Risk of Gastric Cancer in an Eastern Chinese Population
Source: PLoS One. 2012 Nov 15;7(11):e49308. doi: 10.1371/journal.pone.0049308 (PMC3499547; doi:10.1371/journal.pone.0049308)
Supplement: Table S3 — Frequency distribution of demographic characteristics of gastric cancer cases and cancer-free controls. (DOC) [file pone.0049308.s003.doc]

**Table S3.** Frequency distribution of demographic characteristics of gastric cancer cases and cancer-free controls.

| **Variables** | **Cases No. (%)** | **Controls No. (%)** | **P ^a^** |
| --- | --- | --- | --- |
| All subjects | 1125(100) | 1196 (100) |  |
| Age, yr |  |  | 0.557 |
| Range | 21-86 | 22-86 |  |
| Mean ± SD | 58.6 ± 11.4 | 58.6 ± 11.8 |  |
| ≤ 50 | 234 (20.8) | 271 (22.7) |  |
| 51-60 | 383 (34.0) | 384 (32.1) |  |
| 61-70 | 339 (30.1) | 372 (31.1) |  |
| >70 | 169 (15.0) | 169 (14.1) |  |
| Sex |  |  | 0.282 |
| Male | 800 (71.1) | 826 (69.1) |  |
| Female | 325 (28.9) | 370 (30.9) |  |
| Smoking status |  |  | **<0.0001** |
| Never | 686 (61.0) | 610 (51.0) |  |
| Former | 17 (1.5) | 120 (10.0) |  |
| Current | 422 (37.5) | 466 (39.0) |  |
| Drinking status |  |  | **0.008** |
| Yes | 270 (24.0) | 345 (28.8) |  |
| No | 855 (76.0) | 851 (71.2) |  |
| Tumor site |  |  |  |
| GCA | 305 (27.1) | — |  |
| NGCA | 820 (72.9) | — |  |
| Clinical stage |  |  |  |
| Stage I+ II | 476 (42.3) | — |  |
| Stage III+ IV | 649 (57.7) | — |  |
| GCA, gastric cardia adenocarcinoma; NGCA, non-gastric cardia adenocarcinoma.  ^a^ Two-sided *χ^2^* test for distributions between cases and controls. | | | |
